# Frailty Assessment in Clinical Practice: Opportunity in the Midst of a Pandemic

**DOI:** 10.3390/geriatrics5040092

**Published:** 2020-11-14

**Authors:** Victoria L. Keevil, Christopher N. Osuafor, Alistair J. Mackett, Richard Biram

**Affiliations:** 1 Department of Medicine for the Elderly, Addenbrooke’s Hospital, Cambridge University Hospitals NHS Foundation Trust, Hills Road, Cambridge CB2 0QQ, UK; christopher.osuafor@addenbrookes.nhs.uk (C.N.O.); alistair.mackett@addenbrookes.nhs.uk (A.J.M.); richard.biram@addenbrookes.nhs.uk (R.B.); 2 Department of Medicine, University of Cambridge, Addenbrooke’s Hospital, Hills Road, Cambridge CB2 0QQ, UK; 3 Cambridge Public Health, Forvie Site, University of Cambridge, Cambridge CB2 0SR, UK; 4 Department of Clinical Neurosciences, University of Cambridge, Cambridge CB2 0QQ, UK

**Keywords:** frailty, COVID-19, acute care, education and training

## Abstract

Emerging evidence from studies of older adults hospitalised with COVID-19 suggests that there is a high prevalence of frailty in this patient group. We reflect on the measurement of frailty in older patients hospitalized as an emergency and the translation of frailty from a research to a clinical concept. We consider whether, despite the contemporary challenges in the care of older adults as a result of COVID-19, there are opportunities for care quality improvement during a pandemic.

The brief report by Collins et al. describes a European cohort of older adults hospitalised with COVID-19 and found that 66.9% (n = 854) were frail [1]. We have observed similar results. Frailty, defined as scoring ≥5 on the Clinical Frailty Scale (CFS), was present in 66.4% (n = 142) of older patients (aged ≥65 years) who were hospitalised with COVID-19 at our centre during the ‘first wave’ of the pandemic [2]. We are writing to reflect on these findings and consider the measurement of frailty as part of routine clinical practice, in patients hospitalised as an emergency.

Measurement of frailty using the CFS has been part of the routine assessment of older adults admitted to our centre via the emergency pathway for several years. We previously observed that 40.2% (n = 3299) of patients aged ≥75 years who were admitted as an emergency to our hospital, under any specialty, were frail [3]. Therefore, the prevalence of frailty amongst hospitalised patients with COVID-19 is seemingly higher than anticipated, especially considering the wider age range included in the COVID-19 cohort and the strong association between frailty and older age [1]. This observation is also supported by the wider literature on frailty in hospitalised older adults. Other reports have estimated frailty to be present in 28% of patients ≥65 years old admitted to three acute surgical assessment units across Wales [4]; 40% of patients admitted to an acute geriatric ward at a tertiary hospital in Belgium [5]; 33% of patients aged 70 years and above who were admitted to acute general medical wards in Germany [6]; and 48.8% of all adult inpatients (62% of those were aged ≥65 years) in a tertiary hospital and rehabilitation facility in New Zealand [7]. Currently, there are no systematic reviews or meta-analyses synthesising the range of evidence describing the frailty of hospitalised older adults, and this is an important evidence gap [8]. Such work, along with future reports of frailty and COVID-19, will confirm how the prevalence of frailty in those hospitalised with COVID-19 compares to other older adult inpatient groups and whether our current observations prove correct.

We have previously demonstrated the added value of frailty measurement in the clinical assessment of older patients admitted to our centre. Frailty measurements identified those at risk of negative hospital outcomes, such as inpatient mortality and a prolonged length of stay. It also helped to identify those patients who were most likely to need comprehensive geriatric assessment (CGA), delivered by multi-disciplinary teams experienced in the management of older patients on specialist wards, prior to hospital discharge [3,9,10]. Frailty metrics were better able to predict these outcomes than age or co-morbidity, emphasising the heterogeneity of older adult populations that cannot be captured by age alone [9]. Other work has also associated frailty with conditions such as delirium and deconditioning, common complications of acute hospital admission in older adults [11,12].

These ‘real world’ observations are consistent with the extensive literature that has been published over recent years examining relationships between frailty and late-life health [13,14]. Recognising this, at the start of the pandemic the National Institute for Health and Care Excellence (NICE) recommended measurement of frailty as part of the holistic assessment of older adults presenting with COVID-19, to aid personalised care planning [15]. Collins et al. report a 98.5% completion rate for frailty assessment in their study. However, we note that the frailty assessment was undertaken either by the researcher or the clinical team looking after the patient, and the relative proportions are not reported. Our experience of compliance in a real world clinical setting, is that 72–77% of inpatients undergo routine frailty assessment [3,9]. During the initial period of the pandemic though, we observed a lower compliance rate for patients hospitalised with COVID-19 at our centre. Only 52% were assessed for frailty by their treating team (n = 112) [2]. This was most likely due to the significant changes that clinical services underwent at the start of the pandemic, including redeployment of clinical teams away from their specialty roles in order to meet the new anticipated service demands.

The COVID-19 pandemic has presented notable challenges in the care of older adults but, despite these challenges, opportunities for care quality improvement can still be realised. There has been significant progress in translating frailty from a research to a clinical concept over the last decade [16,17]. However, with the number of older adults rising around the world, providing care for this patient group will require the involvement of clinicians across most specialities, not just those with an interest in the care of older people. However, the lower compliance with frailty assessment at our centre during the pandemic is an indication that non-specialists may still be less familiar with frailty as a clinical concept. A previous scoping review of frailty in acute care settings also found that in non-specialist settings, frailty assessment is often undertaken without using standardised or validated measurement tools, limiting its value [18].

Therefore, given emerging evidence of the high prevalence of frailty amongst older adults hospitalised with COVID-19 and the intense interest in the management of all patients with this disease, we suggest that the pandemic could offer opportunities to increase awareness of frailty across a broad range of healthcare workers from different specialty backgrounds. These opportunities could include the education and training of healthcare staff in the clinical assessment of frailty, and opportunities for innovative service re-organisation, so that services better meet the needs of older patients. Assessment of older adult inpatients for frailty using standardised tools could enable hospitals to identify those patients who may benefit the most from CGA, maximising the effective use of specialist resources and ensuring the best outcomes for patients [19].

In summary, a high proportion of older adults hospitalised with COVID-19 are frail. Recognising frailty on admission to hospital will help healthcare staff to anticipate their care needs, enable early proactive referral to specialist services, support shared decision making between the patient, their carer(s) and healthcare staff, and could improve overall outcomes [19]. As the COVID-19 pandemic continues, we should ensure we do not neglect clinical indicators which may improve the quality of care for older adult inpatients. Additionally, the current focus on an inpatient group with a high proportion of frailty could be utilized to improve awareness and implementation of frailty into routine hospital assessments, as one such clinical indicator.
